# Urinary semaphorin 3A as an early biomarker to predict contrast-induced acute kidney injury in patients undergoing percutaneous coronary intervention

**DOI:** 10.1590/1414-431X20176487

**Published:** 2018-03-01

**Authors:** Li Ning, Zhiguo Li, Dianjun Wei, Haiyan Chen, Chao Yang, Dawei Wu, Yanchun Wang, Jingwei Zhang

**Affiliations:** 1 Department of Clinical Laboratory, The Second Hospital of Tianjin Medical University, Tianjin, China; 2 Department of Nephrology, The Second Hospital of Tianjin Medical University, Tianjin, China; 3 Department of Clinical Laboratory, The First Affiliated Hospital of Xi'an Medical University, Xi'an, Shaanxi Province, China

**Keywords:** Contrast-induced acute kidney injury, Percutaneous coronary intervention, Semaphorin 3A, Neutrophil gelatinase-associated lipocalin, Biomarker

## Abstract

Contrast-induced acute kidney injury (CI-AKI) is a serious complication of diagnostic coronary angiograph and percutaneous coronary intervention (PCI). However, the exact pathophysiological mechanisms underlying CI-AKI development are largely unknown. The present study examined whether urinary semaphorin 3A levels predict the development of CI-AKI in patients undergoing PCI. This study enrolled 168 patients with stable angina undergoing elective PCI. Serial urine samples, obtained at baseline and 2, 6, 12, 24, 36, and 48 h post-PCI were analyzed by semaphorin 3A and neutrophil gelatinase-associated lipocalin (NGAL) ELISA kit. AKI was defined as an increase in serum creatinine beyond 50% according to the RIFLE classification system. Receiver operator characteristic (ROC) curve analyses identified optimal semaphorin 3A and NGAL values for diagnosing CI-AKI. CI-AKI occurred in 20 of 168 patients. There were no significant differences in the baseline clinical characteristics and angiographic findings between non-AKI patients group and AKI patients group. Both urinary semaphorin 3A and NGAL levels significantly increased at 2 and 6 h post-PCI. ROC analysis showed that the cut-off value of 389.5 pg/mg semaphorin 3A at 2 h post-PCI corresponds to 94% sensitivity and 75% specificity and the cut-off value of 94.4 ng/mg NGAL at 2 h post-PCI corresponds to 74% sensitivity and 82% specificity. Logistic regression showed that semaphorin 3A levels at 2 and 6 h post-PCI were the significant predictors of AKI in our cohort. Urinary semaphorin 3A may be a promising early biomarker for predicting CI-AKI in patients undergoing PCI.

## Introduction

Contrast-induced acute kidney injury (CI-AKI) is a serious complication of diagnostic coronary angiography and percutaneous coronary intervention (PCI) (1). CI-AKI often causes adverse clinical outcomes and prolonged hospital stay (2). Studies have proposed that the processes of CI-AKI involve immunologic reactions, ischemic injury, and tubular epithelial cell toxicity (3). Studies also found that there was an increase in hypoxia of the renal medulla and in renal free-radical production through post-ischemic oxidative stress after infusion of contrast medium (4). However, the exact pathophysiological mechanisms underlying CI-AKI development are very complex and largely unknown.

Many risk factors have been suggested to play an important role in the development of CI-AKI. The change of serum creatinine level was well-documented as a risk factor for CI-AKI (5). However, the serum creatinine level does not elevate until glomerular filtration rate (GFR) has decreased by at least 50%, thus, assessment of renal dysfunction according to serum creatinine is not reliable (6). In addition, creatinine clearance value using Cockcroft-Gault formula often overestimates the GFR. As far as we know, the renal markers such as cystatin C, neutrophil gelatinase-associated lipocalin (NGAL), liver fatty acid-binding protein (L-FABP), kidney injury molecule 1 (KIM-1) and interleukin 18 (IL-18) are proposed as potential biomarkers for CI-AKI (7). A recent meta-analysis delivered the evidence for the high predictive power of serum cystatin C assessed within 24 h after renal injury for CI-AKI (8). Tasanarong et al. (9) reported urinary NGAL above the threshold of 117 mg/mL measured after 6 h had a sensitivity of 94%, a specificity of 78%, and an area under the curve (AUC) of 0.84 for predicting CI-AKI. According to a plausible study by Manabe et al. (10), comprising 200 patients, urinary L-FABP greater than 24.5 µg/g creatinine was an independent predictor of CI-AKI development. Studies from Ling et al. (11) showed that urinary IL-18 was significantly increased at 24 h in their CI-AKI group in comparison with patients without this complication. Torregrosa et al. (12) showed that KIM-1 evaluated 12 h after cardiac catheterization in humans showed a good predictive value for CI-AKI.

Recently, semaphorin 3A was identified and validated as a new early diagnostic biomarker for AKI. Semaphorin 3A belongs to the semaphorins family, which are characterized structurally by a conserved ∼400 amino acid sema domain (13). The semaphorins can act as context-dependent chemoattractant, and are identified as collapsing factors and mediators of axon repulsion. Semaphorin 3A acts as a chemorepellent with multiple roles such as cardiac and peripheral vascular patterning, branching morphogenesis, and axon guidance (14,15). Semaphorin 3A signaling is mediated via binding receptor neuropilin 1 and signaling receptors plexinA1 or A3 (16). Semaphorin 3A was found to be expressed in the developing glomerulus as well as the adult podocytes and collecting tubules (17). Studies showed that semaphorin 3A had an inhibitory effect on ureteric bud branching (14). Recent studies have demonstrated the role of semaphorin 3A in acute kidney injury. For instance, semaphorin 3A was found to be a new early diagnostic marker of experimental and pediatric AKI (18). Lewandowska et al. (19) showed that semaphorin 3A predicts the development of AKI in liver transplant patients. In addition, semaphorin 3A inactivation suppresses ischemia-reperfusion-induced AKI (20). However, the pathophysiological role of semaphorin 3A in CI-AKI has not been studied yet. Therefore, the current studies were undertaken to determine whether urinary semaphorin 3A levels predict the development of CI-AKI in patients who underwent PCI.

## Patients and Methods

### Patients

Our study included 168 patients with stable angina undergoing PCI admitted to the Second Hospital of Tianjin Medical University between January 2014 and June 2016. All the patients gave their written informed consent. This study was approved by the Ethical Committee and the Clinical Studies Committee of the Second Hospital of Tianjin Medical University. The exclusion criteria for this study was as follows: 1) acute myocardial infarction or unstable angina; 2) chronic renal failure (serum creatinine greater than 2.0 mg/dL); 3) history of exposure to contrast within 1 week. Before the PCI procedures, we measured serum creatinine, urinary semaphorin 3A, and urinary NGAL. The endpoint used for evaluating the patients was the appearance of AKI, defined as an increase in serum creatinine beyond 50% according to the RIFLE (risk, injury, failure, loss, end-stage renal disease) classification system.

### PCI procedures

PCI procedures were performed using standard techniques. Before angiography, isotonic saline was intravenously infused at a rate of 1 mL·kg^-1^·h^-1^ for 12 h before and after PCI for AKI prevention. Interventional devices were selected according to the operator's preference. Iopamidol 370 was used as radiographic contrast medium, and its volume was highly variable, as needed. The serum creatinine levels were determined from the day prior to the PCI procedure to 6 days after PCI and followed the clinical evaluation of all patients until they were discharged from the hospital. The serum creatinine value measured the day before the intervention was established as the basal creatinine level. Urine samples were collected before PCI procedure, and at 0, 2, 6, 12, 24, 36, and 48 h after PCI procedure for the determination of biomarkers (semaphorin 3A and NGAL).

### Processing of urine samples

Urine samples were centrifuged at 1500 *g* for 15 min at 4°C and the supernatant was stored at -80°C for further analysis.

### Laboratory determinations

The estimated GFR (eGFR) was calculated using the Modification of Diet in Renal Disease equation. Creatinine was measured in serum using standard techniques. Urinary semaphorin 3A was measured by using an enzyme-linked immunosorbent assay kit (Catalogue No. MBS732622, My Biosource, USA) according to a previous study (18). Briefly, semaphorin 3A standard, samples and secondary anti-body HRP conjugate were added to antibody-coated 96-well plates and incubated at 37°C for 1 h. Plates were then washed and color was developed using tetramethylbenzidine substrate, and reaction was arrested by adding sulfuric acid. The color change was measured using a plate reader (BioTek, USA) at a wavelength of 450 nm. Urinary semaphorin 3A concentration was reported as picograms per milligram of urine creatinine. The NGAL urinary concentrations were measured using a commercially available ELISA kit (Antibody Shop, Denmark) following the manufacturer's instructions (9). The inter-assay and intra-assay coefficients of variation for semaphorin 3A and NGAL were less than 5%.

### Statistical analysis

All the statistical analysis was performed by using GraphPad Prism (version 6.0, USA) and SPSS software (USA). All data are reported as means±SE. A two-sample *t*-test or the non-parametric Mann-Whitney U-test was used to compare continuous variables. Chi-square test or Fisher's exact test were performed to compare categorical variables. To measure the sensitivity and specificity of semaphorin 3A and NGAL for the prediction of AKI, receiver-operating characteristic (ROC) curves were generated and the AUC was calculated. The cut-off value was defined as the closest point to sensitivity: specificity = 1.0 on ROC curve. An AUC-ROC value of 0.90-1.0 indicated excellent, 0.8-0.89 good, 0.70-0.79 fair, 0.60-0.69 poor, and 0.50-0.59 indicated no useful value. Univariate and multivariable logistic regression analysis was performed to assess predictors of AKI. P values less than 0.05 were considered to be statistically significant.

## Results

### Clinical characteristics of patients with and without AKI before PCI

Before PCI, we first examined the clinical characteristics of the 168 included patients. As shown in Table 1, there were 116 male and 52 female patients, and the mean age of the whole group was 66.7±3.6 years. Among the included patients, there were 20 patients who developed AKI after PCI procedure, and the AKI rate was 11.9% (20/168). There were no significant differences in the clinical characteristics for age, gender, body mass index, serum creatinine, eGFR, hemoglobin, hemoglobin A1c, left ventricular ejection fraction rate, fasting plasma glucose levels, brain natriuretic peptide level, percentage of hypertension, incidence of diabetes mellitus, and percentage of patients taking diuretics.

**Table 1.** Clinical characteristics of patients with and without acute kidney injury (AKI) before percutaneous coronary intervention.

Variables	Non-AKI (n=148)	AKI (n=20)
Gender (male/female)	100/48	16/4
Age (years)	67.1±3.3	66.2±4.3
BMI (kg/m^2^)	23.2±4.1	24.2±3.2
Creatinine (mg/dL)	0.96±0.07	0.89±0.09
eGFR (mL·min/(1.73 m^2^)	51.2±11.6	47.7±17.1
Hb (mg/dL)	11.8±1.9	12.9±2.5
HbA1c (%)	8.3±1.3	8.0±1.1
LVEF (%)	58±10	51±14
BNP (pg/mL)	256±57	201±43
FPG (mg/dL)	118±43	123±35
Hypertension (%)	102 (68.9%)	20 (100%)
DM (%)	40 (27.0%)	11 (55.0%)
Diuretics (%)	31 (20.9%)	8 (40%)

Data are reported as means±SD or number and percentage. BMI: body mass index; eGRF: estimated glomerular filtration rate; Hb: hemoglobin; HbA1c: hemoglobin A1c; LVEF: left ventricular ejection fraction. BNP: brain natriuretic peptide; FPG: fasting plasma glucose; DM: diabetes mellitus. Statistical analysis was done with the *t*-test or the non-parametric Mann-Whitney U-test for means and the chi-square test or Fisher's exact test for proportions. There were no significant differences between groups.

### Angiographic results and lesion features of patients with or without AKI

The data were compared between the non-AKI group and AKI group, and as shown in Table 2, there were no significant differences in the number of diseased vessels, target vessels, stent diameter, stent length, number of stents used, maximum inflation pressure, and volume of contrast medium.

**Table 2.** Angiographic results and lesion features of patients with or without acute kidney injury (AKI).

Variables	Non-AKI (n=148)	AKI (n=20)
Number of diseased vessels		
1-vessel	46 (31.1%)	2 (10%)
2-vessel	58 (39.2%)	7 (35%)
3-vessel	44 (29.7%)	11 (55%)
Target vessel		
LAD	59 (39.8%)	3 (15%)
LCX	52 (35.1%)	7 (35%)
RCA	37 (25%)	10 (50%)
LMT	4 (2.7%)	0 (0%)
SVG	3 (2.0%)	0 (0%)
Stent diameter (mm)	3.4±0.2	3.1±0.15
Stent length (mm)	22.7±8.7	23.4±7.1
Number of stents used	1.6±0.5	1.5±0.5
Maximum inflation pressure (atm)	15±5.1	14±4.6
Volume of contrast medium	185±65	197±53

Data are reported as means±SD or number and percentage. LAD: left anterior descending coronary artery; LCX: left circumflex coronary artery; RCA: right coronary artery; LMT: left main trunk coronary artery; SVG: saphenous vein graft. Statistical analysis was done with the *t*-test or the non-parametric Mann-Whitney U-test for means and the chi-square test or Fisher's exact test for proportions. There were no significant differences between groups.

### Changes in urinary semaphorin 3A and NGAL concentrations after PCI

**Figure 1.**
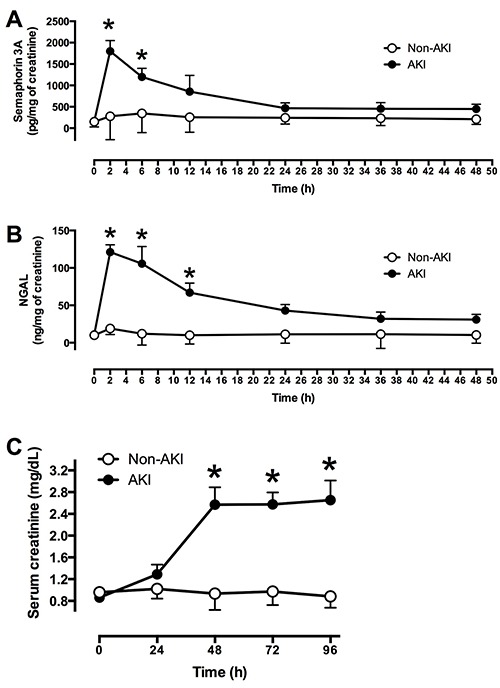
Changes in urinary semaphorin 3A (*A*), urinary neutrophil gelatinase-associated lipocalin (NGAL) (*B*) and serum creatinine concentrations (*C*) at various time points after percutaneous coronary intervention in patients with acute kidney injury (AKI) and without AKI (Non-AKI). Data are reported as means and SD. *P<0.05 between groups (repeated measures two-way ANOVA).

We determined the urinary semaphorin 3A concentrations in patients for up to 48 h post-PCI. As shown in Figure 1A, semaphorin 3A levels were significantly elevated at 2 h and 6 h post-PCI procedure, and peaked at 2 h post-PCI in the AKI patients. Levels of semaphorin 3A between AKI and non-AKI groups were no longer significantly elevated at 12 h post-PCI. In the non-AKI patients, the increase of urinary semaphorin 3A level was much less when compared to AKI patients, and the slight increase was not significantly different from baseline (at t = 0 h) (Figure 1A). In addition, we measured the serum levels of creatinine, which is the gold standard biomarker for AKI, and as shown in Figure 1C, the levels significantly increased at 48, 72, and 96 h after PCI in patients with AKI.

**Figure 2.**
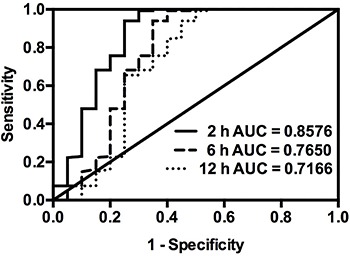
Receiver operating characteristics curves (AUC) of urinary semaphorin 3A at 2, 6, and 12 h post-percutaneous coronary intervention.

Furthermore, conventional ROC curves for AKI *vs* non-AKI were generated for urinary semaphorin 3A at 2, 6, and 12 h post-PCI. The AUCs for the three ROCs curves were 0.8576 (P<0.001), 0.7650 (P<0.001), and 0.7166 (P<0.01), respectively (Figure 2). In addition, the sensitivity and specificity for the semaphorin 3A at optimal concentrations were determined at 2 h post-PCI, and the results showed that the cut-off at 389.5 pg/mg of creatinine corresponds to 94% sensitivity and 75% specificity. Furthermore, we compared the other well-studied early biomarker NGAL for AKI post-PCI. As shown in Figure 1B, the changes of urinary NGAL showed a similar pattern to semaphorin 3A: it significantly increased at 2, 6, and 12 h post-PCI and peaked at 2 h. The ROC curves for AKI *vs* non-AKI were also generated for urinary NGAL at 2, 6, and 12 h, and the AUCs for the three ROCs were 0.632 (P<0.05), 0.657 (P<0.05), and 0.619 (P<0.05), respectively. Further analysis showed that the cut-off value of at 94.4 ng/mg of creatinine for ROC at 2 h post-PCI corresponds to 74% sensitivity and 82% specificity. In addition, the AUC for combined semaphorin 3A and NGAL at 2 h post-PCI was also calculated, and the results showed that the simultaneous occurrence of the 2 urinary biomarkers above the designated threshold did not improve the AUC for the prediction of AKI (Table 3).

**Table 3.** Predictive features for various combinations of biomarkers at 2 h post-surgery.

Biomarker or combination	AUC	Sensitivity	Specificity
SEMA (389.5 pg/mg of creatinine)	0.857	0.94	0.75
NGAL (94.4 ng/mg of creatinine)	0.632	0.74	0.82
SEMA + NGAL	0.733	0.81	0.78

AUC: area under the ROC curve; SEMA: semaphorin 3A; NGAL: neutrophil gelatinase-associated lipocalin.

### Association between semaphorin 3A and clinical characteristics of patients undergoing PCI

Univariate logistic regression identified that higher concentrations of semaphorin 3A at 2 and 6 h post-PCI were significantly associated with higher odds of AKI. A further stepwise logistic regression analysis was used to determine the most efficient model given a set of potential variables for predicting AKI. Potential variables for this model included gender, age, body mass index, surgery time, serum creatinine, eGFR, hemoglobin, hemoglobin A1c, left ventricular ejection fraction rate, fasting plasma glucose levels, brain natriuretic peptide level, percentage of hypertension, the incidence of having diabetes mellitus, and percentage of patients taking diuretics. The final model showed that semaphorin 3A levels at 2 and 6 h post-PCI were the significant predictors of AKI in our cohort (Table 4).

**Table 4.** Prediction of acute kidney injury in univariate and multivariate analysis.

Predictor (SEMA >389.5 pg/mg creatinine)	Univariate		Multivariate	
	Odds ratio (95%CI)	P value	Odds ratio (95%CI)	P value
2 h post-surgery	4.67 (2.55–8.97)	0.0017	3.55 (1.56–7.83)	0.014
6 h post-surgery	3.21 (1.78–5.98)	0.0029	2.12 (1.11–5.23)	0.021
12 h post-surgery	1.12 (0.76–3.14)	NS		

SEMA: semaphorin 3A; NS: not significant.

## Discussion

This study demonstrated for the first time that urinary semaphorin 3A is an early predictive biomarker of CI-AKI. Patients undergoing PCI that had CI-AKI showed significantly elevated levels of urinary semaphorin 3A within the first 2 h after PCI, which is much earlier than the rise in serum creatinine by 48–72 h. However, the role of semaphorin 3A in kidney pathophysiology is unknown. Semaphorin 3A is known to have anti-angiogenic effect, but whether semaphorin 3A regulates angiogenesis has not been studied. Since semaphorin 3A was found to regulate cell migration and adhesion, it is likely that it may regulate epithelial cell proliferation and migration, which often occurs immediately after AKI. In animal studies, semaphorin 3A was found to be localized in distal tubules of the kidney and its levels increased within 3 h after reperfusion of the kidney whereas serum creatinine was significantly raised at 24 h (18). In a more detail animal study, genetic inactivation of semaphorin 3A and pharmacologically based inhibition of semaphorin 3A receptor protected mice from ischemia-reperfusion-induced AKI, and semaphorin 3A was suggested to exacerbate AKI via promoting inflammation and epithelial cell apoptosis (20). Therefore, we may perform future animal studies to look into the mechanistic role of semaphorin 3A in CI-AKI development.

NGAL is a 21-kDa, calyx-shaped protein engaged in innate nonspecific immunity mechanisms against bacterial infections and secreted via toll-like receptor activation (21). Various studies have suggested urinary NGAL as a powerful diagnostic tool for CI-AKI. Tasanarong et al. (9), reported urinary NGAL above the threshold of 117 mg/mL measured after 6 h had a sensitivity of 94%, a specificity of 78% and an area under the curve (AUC) of 0.84 for predicting CI-AKI in the patients undergoing elective cardiac catheterization. Further study showed that both urinary and serum NGAL concentrations at 2 and 4 h after PCI, respectively, predicted CI-AKI development (22). In the present study, we used NGAL as a reference biomarker for CI-AKI in comparison with the predictive effect of semaphorin 3A in CI-AKI. We found that both urinary semaphorin 3A and NGAL levels increased significantly at 2, 6, and 12 h after PCI. Further ROC results showed that the AUC of ROC for semaphorin 3A at 2 h after PCI was higher than that of NGAL. ROC analysis of semaphorin 3A at 2 h after PCI showed better predictive sensitivity and specificity when compared to NGAL, which suggests that semaphorin 3A may be a more powerful predictive factor of CI-AKI development in patients undergoing PCI. Indeed, semaphorin 3A has been shown to be a promising biomarker for AKI. Semaphorin was found to predict the development of AKI in liver transplant patients, and the AUC of ROC for semaphorin 3A at 2 h after surgery was 0.631 with an optimal sensitivity of 57% and specificity of 77% (19). In the pediatric AKI, the AUC of ROC for semaphorin 3A at 2 h after surgery was 0.880 with an optimal sensitivity of 81% and specificity of 94% (18). The present study also found comparable results, in which the AUC of ROC for semaphorin 3A at 2 h after PCI was 0.8756 with an optimal sensitivity of 75% and specificity of 82%. Our results may suggest that semaphorin may be a reliable early biomarker to predict CI-AKI in patients undergoing PCI.

The present study has several strengths. We identified a new biomarker for CI-AKI and validated it in human samples. In our study, all subjects started with normal kidney function and low levels of semaphorin in the urine. Our study also allowed for the temporal definition of changes in semaphorin 3A concentrations in urine after PCI, and a direct comparison with changes in serum creatinine, which is the gold standard for definition of AKI. We also adjusted for the concentration by correcting urinary semaphorin 3A concentrations with urinary creatinine. However, this study also has limitations. It is a single-center pilot study of patients with stable angina undergoing PCI. Thus, these results should be validated in a larger population and multi-center study. Recent studies also showed that renal markers such as cystatin C, NGAL, L-FABP, KIM-1 and IL-18 are proposed as potential biomarkers for CI-AKI. However, all biomarkers have individual strengths and weaknesses. Given the multifactorial etiologies of AKI, it is unlikely that any single biomarker will suffice. In the future, using a combination of biomarkers might be more accurate for the prediction of CI-AKI.

In conclusion, the present study identified semaphorin 3A as biomarkers for CI-AKI development in patients undergoing PCI. This study may prompt further research into the use of semaphorin 3A along with other currently well-known biomarkers to detect CI-AKI prior to therapeutic strategies in clinical studies. Therapeutic studies based on diagnosis from these biomarkers can be promising in the future.
